# Relationship of cardiometabolic parameters in non-smokers, current smokers, and quitters in diabetes: a systematic review and meta-analysis

**DOI:** 10.1186/s12933-016-0475-5

**Published:** 2016-11-24

**Authors:** Debasish Kar, Clare Gillies, Francesco Zaccardi, David Webb, Samuel Seidu, Solomon Tesfaye, Melanie Davies, Kamlesh Khunti

**Affiliations:** 1 Diabetes Research Centre, University of Leicester, Leicester General Infirmary, Gwendolen Road, Leicester, LE5 4AW UK; 2 Academic Unit of Diabetes and Endocrinology, University of Sheffield, Sheffield, UK; 3 Derbyshire Community Health Services NHS Foundation Trust, Castle Street Medical Centre, Castle Street, Bolsover, Chesterfield, Derbyshire, UK

**Keywords:** Smoking, Smoking cessation, Glycosylated haemoglobin (HbA1c), Diabetes, Low density lipoprotein (LDL) and high density lipoprotein (HDL) cholesterol: blood pressure (systolic and diastolic—SBP and DBP)

## Abstract

**Background:**

Smoking is associated with increased macrovascular and microvascular complications in people with diabetes. In addition to other concomitant vascular perturbations, it also seems to influence the cardiometabolic parameters, which may partly explain the accelerated rate of vascular complications in smokers with diabetes. While smoking cessation is advocated as a universal component of the management of diabetes, there is some anecdotal evidence that HbA1c could increase following smoking cessation. The aim of this review is to explore the relationship between smoking and its cessation on cardiometabolic parameters in diabetes.

**Methods:**

Searches were conducted on Medline, EMBASE and CINAHL up to March 2016. After screening 6866 studies (Additional file 1), 14 observational studies with a total of 98,978 participants’ with either type 1 or type 2 diabetes were selected for review. Narrative synthesis and meta-analyses were carried out to explore the relationship between smoking and its cessation.

**Results:**

Meta-analysis showed that the pooled mean difference of HbA1c between non-smokers and smokers was −0.61% (95% CI −0.88 to −0.33, p < 0.0001). The difference in LDL cholesterol between non-smokers and smokers was −0.11 mmol/l (95% CI −0.21 to −0.01, p = 0.04). The difference in HDL cholesterol between non-smokers and smokers was 0.12 mmol/l (95% CI 0.08–0.15, p < 0.001). However, there was no statistically significant difference in blood pressure between the two groups. The difference in HbA1c between quitters and continued smokers was not statistically significant −0.10% (95% CI −0.42 to 0.21, p = 0.53). However, a narrative synthesis revealed that over a period of 10 years, the HbA1c was comparable between non-smokers and quitters.

**Conclusion:**

Non-smokers have a statistically significant lower HbA1c and more favourable lipid profile compared to smokers. Smoking cessation does not lead to an increase in HbA1c in long-term and may reduce vascular complications in diabetes by its favourable impact on lipid profile.

**Electronic supplementary material:**

The online version of this article (doi:10.1186/s12933-016-0475-5) contains supplementary material, which is available to authorized users.

## Background

Despite an overwhelming body of evidence against smoking and all-out efforts to control tobacco-related harm, globally approximately 6 million deaths are attributed to use of tobacco every year [1]. If the current trend of smoking continues, the World Health Organisation (WHO) estimates that by 2030, the annual death toll will rise to over 10 million [2]. Smoking appears to positively contribute to glucolipotoxicity and insulin resistance, which are the hallmarks of diabetes. Nicotine and the free radicals in cigarettes have been linked to accelerated β-cell apoptosis and impedance of intracellular GLUT-4 mobilisation, which may feed into hyperglycaemia associated with diabetes [3–5]. A number of studies have demonstrated that smoking is associated with increased cardiovascular mortality in people with diabetes [6, 7]. However, it is not entirely clear whether this increased mortality in smokers is due to atherogenic metabolic profile or due to the direct toxic effects of nicotine and other toxic substances in cigarettes on the cardiovascular milieu.

The European Association for Study of Diabetes (EASD) and the American Diabetes Association (ADA) recommend smoking cessation as an integral component of the management of diabetes [8]. Other international and national guidelines, including the World Health Organization (WHO), the National Institute for Health and Care Excellence (NICE) and the Scottish Intercollegiate Guidelines Network (SIGN) in the UK, have published similar recommendations [9, 10]. Despite multiple recommendations, the prevalence of smoking in people with and without diabetes remains comparable [11]. One of the commonest arguments against quitting in people with diabetes is the risk of weight gain and worsening glycaemic control after quitting [12, 13]. Some studies have demonstrated a positive correlation between weight gain and increased HbA1c after quitting [14, 15]. Interestingly, a number of studies also demonstrated a positive correlation between smoking cessation and developing diabetes suggesting that smoking cessation may have a detrimental impact on glucose metabolism [16–18]. Due to the risk of weight gain and the potential risk of worsening glycaemic control, there is a significant anxiety about the benefit of smoking cessation in people with diabetes [19, 20]. The aim of this systematic review and meta-analysis was to explore the precise relationship of the cardiometabolic profiles in smokers, non-smokers, and quitters with diabetes.

### Definition of outcomes and comparisons

For this study, HbA1c was defined as the average plasma glucose level over the preceding 3 months period, measured by high-performance liquid chromatography (HPLC) and expressed as the ratio to total haemoglobin in percentage. NICE recommends the target range for HbA1c for people with diabetes, taking into consideration other vascular risk factors and co-morbidities, to be 6.5–7.5%. In this study for lipid profiles we focused on high-density lipoprotein cholesterol (HDL-C) and low-density lipoprotein cholesterol (LDL-C). HDL-C is the cardioprotective cholesterol, which plays a pivotal role in removing the harmful fat particles from the circulation and protects from cardiovascular events. The normal range of HDL-C is 1.3–1.5 mmol/l. LDL-C, on the other hand, is atherogenic cholesterol, which causes atherosclerosis and thromboembolic events (normal range of LDL-C is 2.59–3.34 mmol/l). Smokers were defined as self-reported smokers who smoked cigarettes for at least 12 months, without any biochemical verifications. Non-smokers were defined as people who never smoked cigarettes. Quitters were former cigarette smokers who gave-up after smoking for at least 12 months, and remained abstinent for at least 12 months. The rationale for using these criteria was, in order to observe any meaningful change in cardiometabolic parameters, it was deemed to be a bare minimum length of time. People with diabetes was defined as those individuals who had an HbA1c of ≥6.5% or being treated with glucose lowering medications, irrespective of their HbA1c value.

## Methods

### Search strategy and selection criteria

In order to explore the relationship of HbA1c, lipid profiles and blood pressure in smokers vs. non-smokers and smokers vs. quitters, we carried out a comprehensive database search. Prior to embarking upon a full search, we carried out a scoping search on Cochrane Library (4th March 2016, using the search terminology diabetes AND smoking AND glycaemia and/or lipid profile and/or blood pressure). We also searched PROSPERO, Pubmed, and Google Scholar but did not find any published or on-going reviews with similar aims. We assessed the titles of the articles for suitability for inclusion using the acronym PECO—population (people with diabetes); exposure (cigarette smoking); comparison (not smoking and/or quitting) and outcome (HbA1c, lipid profile and blood pressure).

**Fig. 1:**
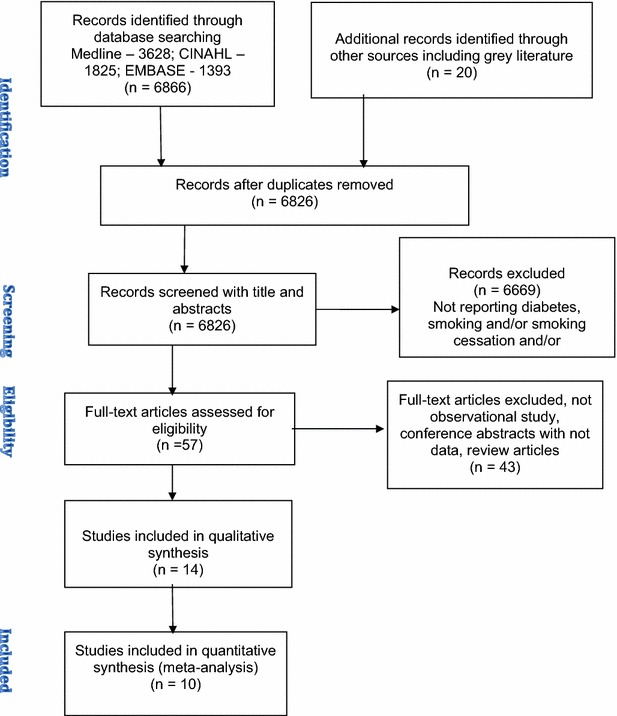
PRISMA flow chart

The search was conducted on EMBASE (1978 to March 2016), and CINAHL (1981 to March 2016) (Additional file 1) and OVID/Medline (from 1946 to March 2016) (Additional file 2). The focus of our search was to identify three separate themes of medical subject headings (MeSH): smoking status (current smokers, never smokers and quitters), type 1 and type 2 diabetes and HbA1c, lipid profile and/or blood pressure. The three themes were then combined using the Boolean operator “and”, imposing no restrictions on type of diabetes or age of the participating subjects to capture as much data as possible. The detailed search strategy using PRISMA flow chart is shown in Fig. 1. In addition, a hand search was carried out on the bibliography of a number of review articles, to identify any relevant publications [21, 22]. Review protocol, inclusion, and exclusion criteria were agreed with the review team and were published in the International Prospective Register of Systematic Review. This systematic review and meta-analysis were reported following the PRISMA and MOOSE guideline [23, 24].

#### Inclusion criteria

1. Participants with either type 1 or type 2 diabetes.
2. HbA1c and/or lipid profiles and/or blood pressure were reported as the outcomes.
3. Participants were classified as smokers, non-smokers or quitters.
4. Studies were reported in English language.
5. Newcastle-Ottawa quality scale for observational studies score >5.

#### Exclusion criteria

1. Any other types of diabetes apart from type 1 or type 2 diabetes.
2. Review articles and meeting abstracts without any relevant data.
3. Studies not in English language.
4. Smoking status not recorded or smokers of less than 12-months duration or quitters not abstinent for at least 12 months.
5. Newcastle-Ottawa quality scale for observational studies score <5.
6. Reported association without any retrievable data.

### Study selection

Two investigators (DK and FZ) assessed the eligibility of the studies using the inclusion and exclusion criteria. A third investigator (DW) resolved any discrepancies in the opinions of the two other investigators. DK and CG independently extracted the data and came to a consensus concerning data accuracy.

### Data extraction and quality assessment

We made an a priori decision to select observational cross-sectional, prospective and retrospective studies. As the heterogeneity between observational studies is likely to be high, random effects models were fitted for the meta-analyses. We extracted the data using a pre-designed data extraction template describing the study characteristics and the reported results. All the variables were converted to the same units i.e. for HbA1c described in mmol/l was converted to % using the standard conversion chart; lipid profiles reported in mg/dl were converted to mmol/l using the formula (mg/dl = ×0.0555 = mmol/l) and blood pressure were reported as mm of Hg. If the information was unavailable or unclear from the published literature, we contacted the study authors for clarification. For the data quality of individual studies, we used the Newcastle-Ottawa quality assessment scale used for observational studies [25].

### Statistical analysis

Narrative synthesis, meta-analysis and meta-regression (Additional file 3) were conducted depending on the availability of suitable data. Narrative synthesis is primarily based on the descriptive analysis of individual studies and is a useful tool to summarise the findings, even if the results cannot be pooled together for meta-analysis. A narrative synthesis is an integral component of any systematic review irrespective of whether a meta-analysis can be carried out or not. Meta-analysis, on the other hand, is a comprehensive method of statistical analysis by collating data from a number of studies to give a pooled estimate of the effect size. Meta-regression models were fitted to assess the relationship between the study effect size and study level covariates, allowing heterogeneity between study results to be better understood.

For this study, the significance level for heterogeneity was set at p < 0.1 and for overall effect size p < 0.05. Cochrane Review Manager Version 5 was used for the meta-analysis and Strata 14 was used to fit the meta-regression models. Heterogeneity was assessed using both the Chi^2^ test and by evaluating the I^2^ value, which quantifies the percentage of total variation across studies that is due to heterogeneity rather than sampling error [26]. Univariate meta-regression models were fitted to assess if the effect size was significantly associated with mean age of the study population, gender (% male), whether the study was conducted on adults (>22 years) or adolescents (18–22 years), whether the participants had type 1 or type 2 DM, study design (cohort or cross-sectional) and mean years smoked.

## Results

In this systematic review, we conducted two types of analyses (narrative synthesis and meta-analysis), to compare two groups of populations (smoker vs. non-smokers and smokers vs. quitters), on three types of outcomes (HbA1c, lipid profiles and blood pressure). In addition, we also conducted meta-regression analysis to explore the association between study effect size and study level covariates such as age, gender, whether the participants were adults or adolescents, types of diabetes, study design and duration of smoking.

Using the agreed search terms, we identified 6866 articles on Medline, EMBASE, and CINAHL (Fig. 1). We reviewed 57 full-text articles, 16 of which met the inclusion criteria for the review and meta-analysis. Out of the 16 studies, 2 were excluded from the review; one of them was due to poor data quality [24] and the other one was because of participants’ resuming smoking within 12 months of quitting [25]. All the 14 studies were included for narrative synthesis. Out of the 14, 12 studies were cross-sectional while one study was a retrospective and another was a prospective cohort study. 10 studies were included for meta-analysis and the rest were excluded due to insufficient data. For smokers and non-smokers, we identified 10 studies for the outcome of HbA1c, 6 for lipid profile and 8 studies for blood pressure.

For smokers and quitters, only 4 out of 5 studies could be used for meta-analysis for the outcome of HbA1c. There was not enough data to pool the results for lipid profile and blood pressure for meta-analysis in this comparison groups. Full details of the study characteristics are summarised in Tables 1, 2, 3 and 4. Meta-regression data and results are shown in Tables 5, 6, 7, 8, 9, 10 and 11.

**Table 1:** Study characteristics of smokers and non-smokers

Study	Country	No of participant	Intervention/control		Age/sex		Included/excluded from meta-analysis Study design
			Smoker	Non-smoker	Smoker	Non-smoker	
Reynolds et al. [42] T2DM	USA	414	66	348	18.6 39.4% M	15.6 35.6% M	Included Cross sectional study
Reynolds et al. [42]T1DM	USA	2327	203	2124	18.3 50.7% M	14.2 50.5% M	Included Cross sectional study
Hofer et al. [43] T1DM	Austria and Germany	27,561	4051	23,510	13.66 21.6% M	13.66 78.4% M	Included Cross sectional study
Thomas et al. [44] T2DM	China and Hong Kong	496	196	300	53.5 100% M	53 100% M	Included Cross sectional study
Schwab et al. [45] T1DM	Germany	92	19	73	15.9 100% F	11.7 100% F	Excluded (skewed data) Cross sectional study
Wakabayashi et al. [46] T1 and T2 DM	Japan	2563	1332	1231	52.15	54.7	Included Cross sectional study
Hanesn et al. [47] T1DM	Denmark	32	16	16	31 87.5% M	31 87.5% M	Included Cross sectional study
Nilsson et al. [48] T1DM	Sweden	11,513	1646	9867	41.3 47% M	40.8 55% M	Included Cross sectional study
Nilsson et al. [48] T2DM	Sweden	40,648	4512	36,136	61.1 61% M	68.4 53% M	Included Cross sectional study
Gerber et al. [49] T2DM	UK	763	603	160	35.1 71.3% M	36.1 52.1% M	Included Prospective cohort study
Ohkuma et al. [50] T2DM	Japan	1184	679	505	65.6	61.2	Included Cross sectional study

**Table 2:** Study characteristics of smokers and quitters

Study	Country	No of participants	Exposure/control		Age/sex		Included or excluded from meta-analysis Study design
			Smoker	Quitter	Smoker	Quitter	
Lycett et al. [51] T2DM	UK	10,692	7561	3131	61.6 (12.1) 58.4% M	61.6 (11.3) 64.4% M	Included Retrospective cohort study
Iino et al. [52] T2DM	Japan	31	16	15	60.1 (2.5) 81.25% M	62.7 (2.5) 80% M	Excluded (subjects started smoking within 12 months) Prospective cohort study
Reynolds et al. [42] T2DM	USA	125	66	59	18.6 (2.5) 39.4%	17.9 (2.4) 47.5% M	Included Cross sectional study
Reynolds et al. [42] T1DM	USA	412	203	209	18.3 (2.2) 50.7% M	17.7 (2.7) 47.4% M	Included Cross sectional study
Ohkuma et al. [50] T2DM	Japan	2490	679	1306	61.2 (10)	66.9 (9.2)	Included Cross sectional study

**Table 3:** Cardiometabolic parameters of smokers and non-smoker of included studies

Study	Cardiometabolic parameters									
	Smokers					Non-smokers				
	HbA1c (%) Mean (SD)	HDL mmol/l Mean (SD)	LDL mmol/l Mean (SD)	SBP mm of Hg Mean (SD)	DBP mm of Hg Mean (SD)	HbA1C (%) Mean (SD)	HDL mmol/l Mean (SD)	LDL mmol/l Mean (SD)	SBP mm of Hg Mean (SD)	DBP mm of Hg Mean (SD)
Reynolds et al. [42] (T2DM)	8.85 (±2.89)	2.23 (±0.59)	6.23 (±2.06)	120.8 (±11.8)	74.8 (±10.2)	7.77 (±2.37)	2.36 (±0.59)	5.79 (±1.75)	116.4 (±12.9)	72.2 (±10.8)
Reynolds et al. [42] (T1DM)	9.16 (±2.08)	2.84 (±0.71)	5.82 (±1.76)	110.7 (±10.9)	71.1 (±9.8)	8.19 (±1.63)	2.98 (±0.69)	5.39 (±1.49)	105.6 (±10.9)	66.9 (± 9.6)
Hofer et al. [43]	9.1 (±1.91)	3.35 (±1.84)	5.24 (±3.56)	116.8 (±14)	68.2 (±10.82)	8.0 (±3.07)	3.53 (±2.76)	5.26 (±5.52)	117.37 (±19.93)	67.6 (±15.33)
Thomas et al. [44]	8.2 (±2.0)	1.12 (±0.31)	3.6 (±0.9)	131 (±20)	82 (±12)	7.6 (±1.8)	1.20 (±0.30)	3.5 (±0.8)	135 (±20)	82 (±12)
Schwab et al. [45]	9.3 (9.0–10.9)	3.22 (3.05–3.44)	6.16 (5.94–6.38)	125 (120–130)	69 (67–70)	7.4 (7.3–7.5)	3.33 (3.16–3.38)	4.99 (4.77–5.16)	120 (120–120)	125 (120–130)
Wakabayashi et al. [46]	7.75 (±1.59)	1.30 (±0.35)	3.12 (±0.87)	Not reported	Not reported	7.48 (±1.52)	1.39 (±0.36)	3.04 (±0.81)	Not reported	Not reported
Nilsson et al. [48]	8.7 (±1.2)	Not reported	Not reported	127 (±11.0)	73 (±7.0)	8.1 (±1.4)	Not reported	Not reported	128 (±13.0)	75 (±10.0)
Nilsson et al. [48]	7.67 (1.44)	Not reported	Not reported	130 (17.9)	73.5 (8.7)	7.21 (1.34)	Not reported	Not reported	129.3 (16.7)	74.9 (8.9)
Ohkuma et al. [50]	6.65 (1.50)	Not reported	Not reported	142.2 (19.3)	79.0 (9.6)	6.44 (1.39)	Not reported	Not reported	146.2 (19.4)	79.1 (9.6)
Hansen et al. [47]	8.7 (1.2)	Not reported	Not reported	127 (11)	73 (7.0)	8.1 (1.4)	Not reported	Not reported	128 (13)	75 (10)
Gerber et al. [49]	9.0 (2.3)	1.53 (0.46)	2.78 (1.00)	127.6 (18.6)	76.8 (12.3)	8.1 (2.0)	1.68 (0.51)	2.7 (0.92)	126.2 (15.7)	77.9 (10.3)

**Table 4:** Cardiometabolic parameters of smokers and quitter of included studied

Study	Cardiometabolic parameters									
	Smokers					Quitters				
	HbA1C (%)	HDL mmol/l	LDL mmol/l	SBP mm of Hg	DBP mm of Hg	HbA1C (%)	HDL mmol/l	LDL mmol/l	SBP mm of Hg	DBP mm of Hg
Lycett et al. [51]	7.7 (1.66)	Not reported	Not reported	Not reported	Not reported	7.9 (1.64)	Not reported	Not reported	Not reported	Not reported
Reynolds et al. [42]	9.16 (2.08)	2.84 (0.71)	5.82 (1.76)	110.7 (10.9)	71.1 (9.8)	8.89 (2.07)	2.94 (0.76)	5.94 (1.72)	109.8 (10.6)	71.2 (9.7)
Reynolds et al. [42]	8.85 (2.89)	2.23 (0.59)	6.23 (2.06)	120.8 (11.8)	74.8 (10.2)	8.23 (2.89)	2.94 (0.76)	5.97 (1.86)	117.2 (11.3)	74.2 (10.2)
Ohkuma et al. [50]	7.47 (1.04)	Not reported	Not reported	Not reported	Not reported	7.28 (1.08)	Not reported	Not reported	Not reported	Not reported

**Table 5:** Data used in meta-regression analyses

Study	Mean age	Participants age	Type of diabetes	% Male	Study design	Duration smoked (mean years)	Pack years	Years stopped
Gerber et al. [49]	35.9	Over 16	1	56	Cohort	Not reported	21.6	Not reported
Hansen et al. [47]	31.9	Over 16	1	59	Cross-sectional	15.5	Not reported	Not reported
Hofer et al. [43]	13.7	Under 22	1	52.5	Cross-sectional	Not reported	Not reported	Not reported
Lycett et al. [51]	61.6	Over 16	2	60.2	Cohort	Not reported	Not reported	Not reported
Nilsson et al. [48]	40.9	Over 16	1	53.8	Cross-sectional	Not reported	Not reported	Not reported
Nilsson et al. [48]	67.6	Over 16	2	54	Cross-sectional	Not reported	Not reported	Not reported
Ohkuma et al. [50]	65.1	Over 16	2	100	Cross-sectional	41.6	43.8	17.6
Reynolds et al. [42]	14.8	Under 22	1	50.2	Cross-sectional	4.5	Not reported	Not reported
Reynolds et al. [42]	16.3	Under 22	2	37.6	Cross-sectional	2.1	Not reported	Not reported
Thomas et al. [44]	53.2	Over 16	2	100	Cross-sectional	Not reported	Not reported	Not reported
Wakabayashihi et al. [46]	53.4	Under 22	1 and 2	100	Cross-sectional	Not reported	Not reported	Not reported

**Table 6:** Meta-regression analysis for difference in HbA1c between smokers and non-smokers

Covariate	N studies	Coefficient (95% CI)	p value
Mean study age	10	0.02 (0.01, 0.02)	<*0.001*
Study age criteria (under 22 or over 18)	10	0.36 (−0.16, 0.89)	*0.016*
Type I or Type II diabetes	9	0.43 (−0.07, 0.93)	0.080
Male (percent)	10	0.01 (−0.00, 0.02)	0.101
Study design	10	−0.33 (−1.31, 0.65)	0.461
Duration smoked	4	0.021 (0.004, 0.038)	*0.034*

The italics values were to indicate statistical significance. A value of <0.05 was statitically significant

**Table 7:** Meta-regression analysis for difference in HDL between smokers and non-smokers

Covariate	N studies	Coefficient (95% CI)	p value
Mean study age	6	−0.002 (−0.004, 0.000)	0.052
Study age criteria (under 22 or over 18)	6	0.019 (−0.144, 0.016)	0.697
Type I or Type II diabetes	5	−0.08 (−0.19, 0.04)	0.119
Male (percent)	6	−0.001 (−0.003, 0.000)	0.053
Study design	6	−0.035 (−0.123, 0.193)	0.569
Duration smoked	6	Not enough observations	N/A

**Table 8:** Meta-regression analysis of LDL between smokers and non-smokers

Covariate	N Studies	Coefficient (95% CI)	p value
Mean study age	6	0.003 (−0.009, 0.014)	0.052
Study age criteria (under 22 or over 18)	6	0.067 (−0.365, 0.499)	0.691
Type I or type II diabetes	5	−0.05 (−0.74, 0.63)	0.825
Male (percent)	6	0.002 (−0.006, 0.010)	0.522
Study design	6	0.062 (−0.483, 0.608)	0.767
Duration smoked	Not reported	N/A	N/A

**Table 9:** Meta-regression analysis for difference in SBP between smokers and non-smokers

Covariate	N studies	Coefficient (95% CI)	p value
Mean study age	8	0.129 (0.017, 0.241)	*0.30*
Study age criteria (under 22 or over 18)	8	4.51 (−0.68, 9.69)	0.078
Type I or Type II diabetes	8	2.46 (−3.84, 8.75)	0.377
Male (percent)	8	0.12 (−0.04, 0.29)	0.126
Study design	8	0.28 (−9.84, 10.41)	0.948
Duration smoked	3	0.36 (−3.92, 4.63)	0.482

**Table 10:** Meta-regression analysis for difference in DBP between smokers and non-smokers

Covariate	N studies	Coefficient (95% CI)	p value
Mean study age	8	0.08 (−0.02, 0.17)	0.102
Study age criteria (under 22 or over 18)	8	3.47 (0.02, 6.73)	*0.041*
Type I or Type II diabetes	8	*1.26 (*−*3.36, 5.89)*	*0.529*
Male (percent)	8	*0.11 (0.02, 0.20)*	*0.027*
Study design	8	0.57 (−6.55, 7.71)	0.850
Duration smoked	3	0.38 (−3.33, 4.09)	0.410

The italic values were to indicate statistical significance. A value of <0.05 was statitically significant

**Table 11:** Meta-regression analysis for difference in HbA1c between current and quitters

Covariate	N studies	Coefficient (95% CI)	p value
Mean study age	4	0.01 (−0.02, 0.03)	0.432
Study age criteria (under 22 or over 18)	4	0.36 (−1.06, 1.79)	0.391
Type I or type II diabetes	4	0.21 (−1.46, 1.89)	0.640
Male (percent)	4	0.001 (−0.035, 0.037)	0.909
Study design	4	0.40 (0.13, 0.66)	*0.022*
Duration smoked	3	0.003 (−0.063, 0.070)	0.628

The italics values were to indicate statistical significance. A value of <0.05 was statitically significant

### Smokers vs. non-smokers

For the outcome of HbA1c, narrative synthesis was conducted on smokers and non-smokers (n = 87,593). Out of the total study participants, 13,323 (15.21%) were smokers and 74,270 (84.79%) were non-smokers. 8 out of 10 studies specified the gender of the study participants (45.94% male and 54.06% female). 9 out of 10 studies specified the type of diabetes of the study participants of whom 47.41% were people with T1DM and 49.67% were people with T2DM.

#### Narrative synthesis

**Fig. 2:**
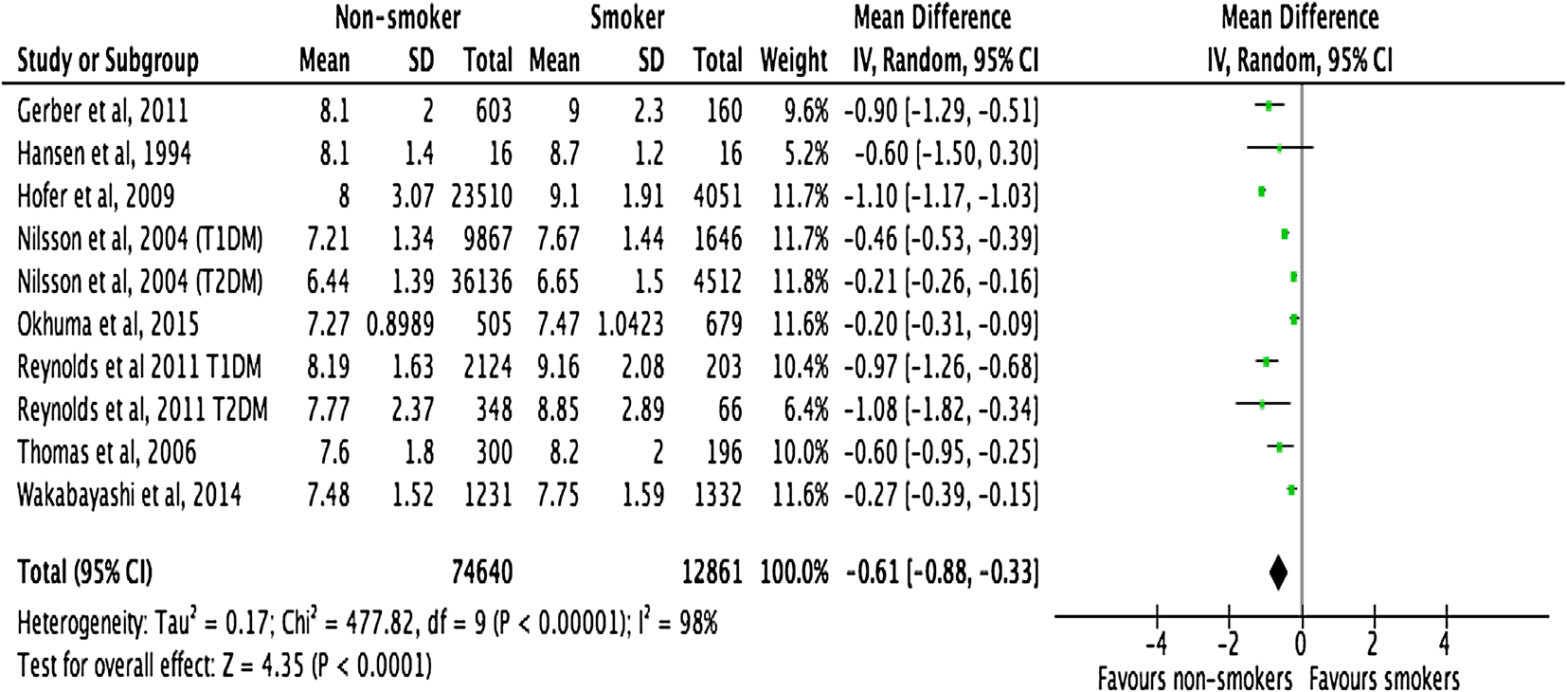
Forest plots. HbA1c (smokers vs. non-smokers)

**Fig. 3:**
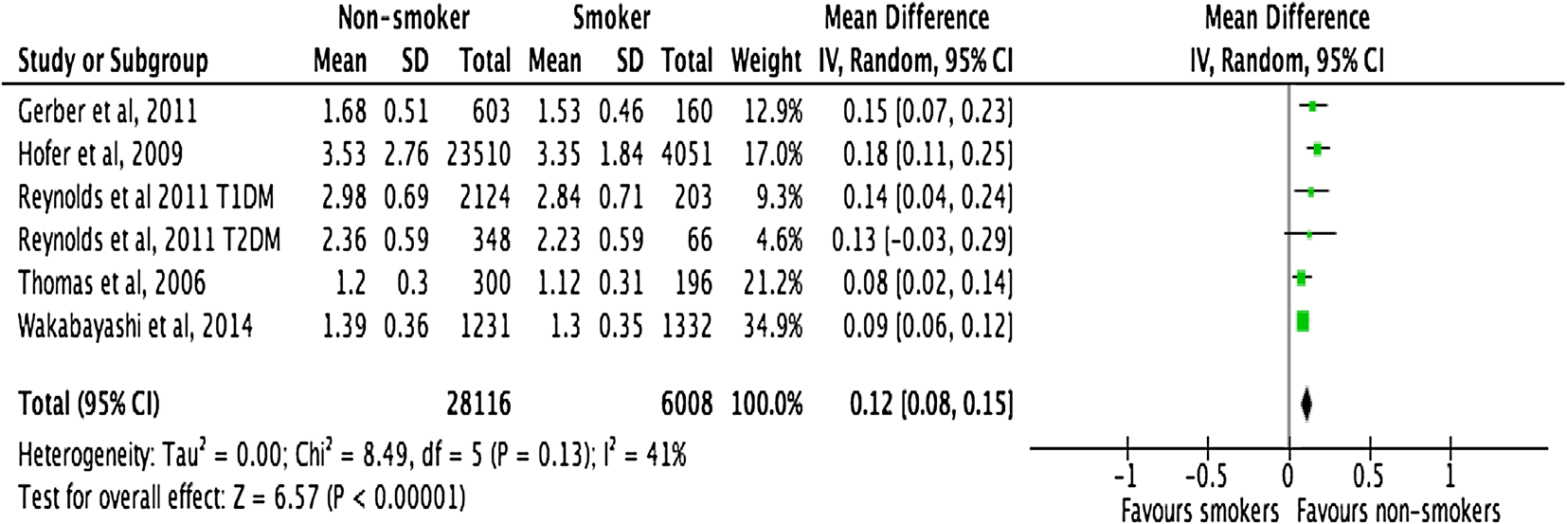
Forest plots. HDL cholesterol (smokers vs. non-smokers)

**Fig. 4:**
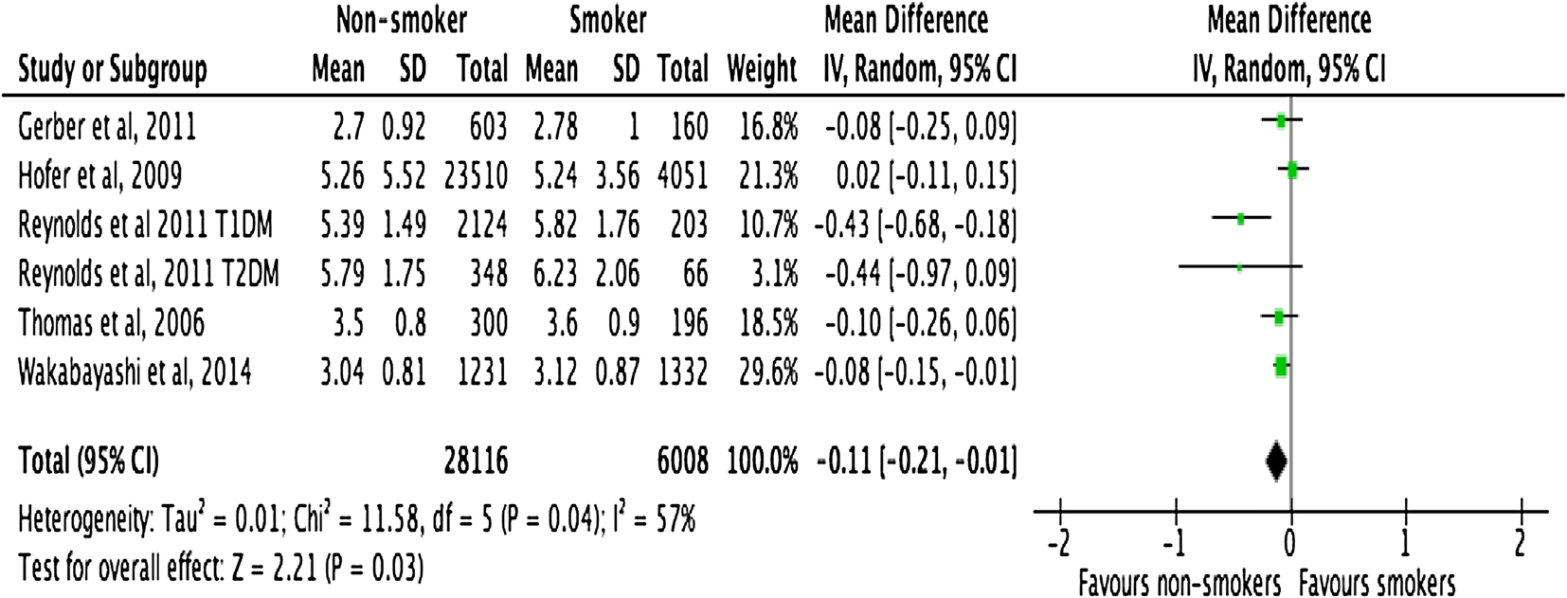
Forest plots. LDL-cholesterol (smokers vs. non-smokers)

**Fig. 5:**
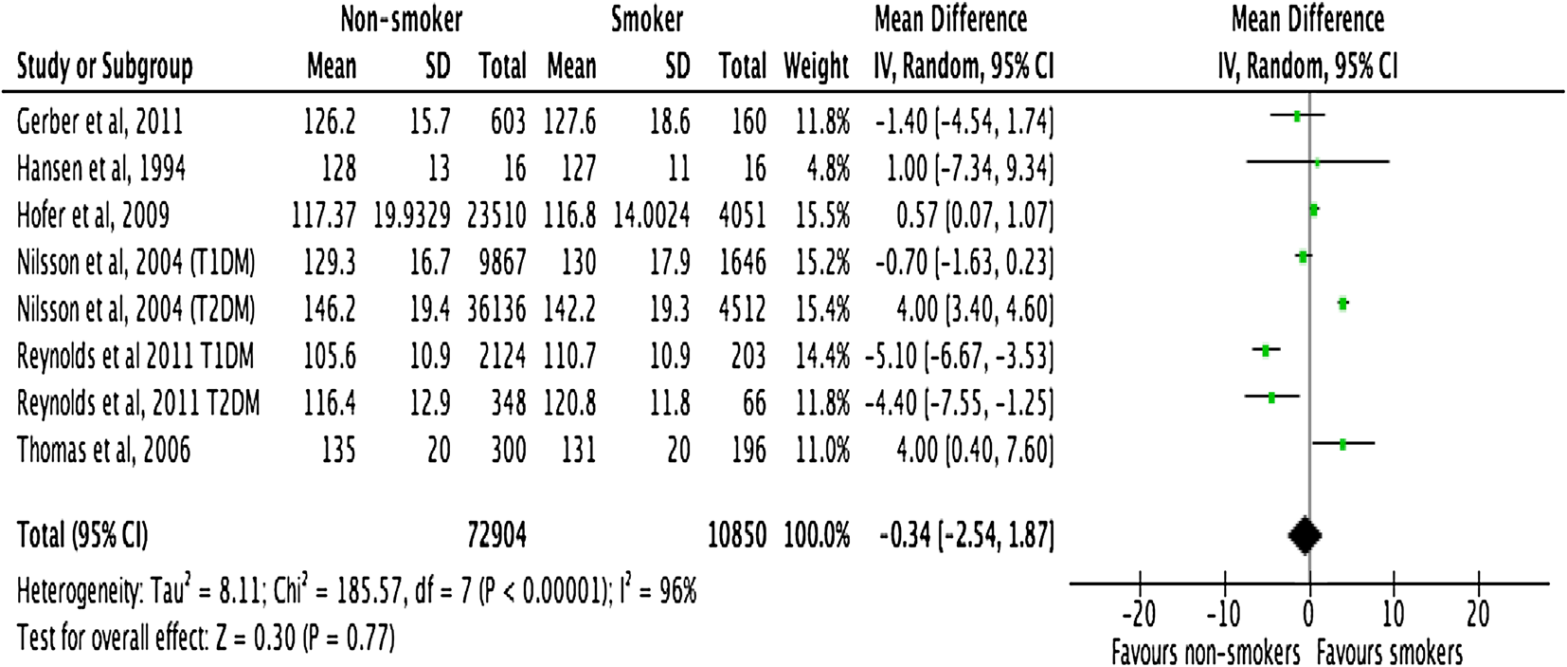
Forest plots. Systolic blood pressure (smokers vs. non-smokers)

**Fig. 6:**
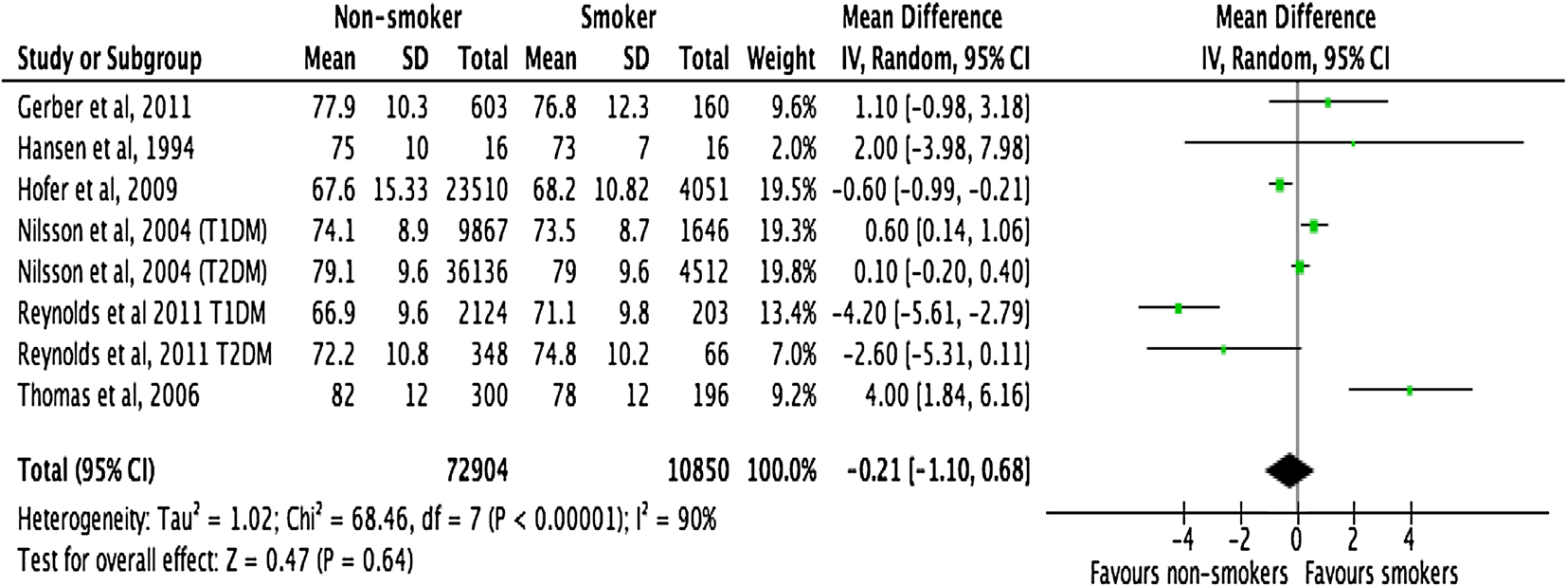
Forest plots. Diastolic blood pressure (smokers vs. non-smokers)

All the included studies demonstrated a close relationship between smoking, HbA1c, and lipid profiles in people with T1DM and T2DM (Figs. 2, 3, 4). However, there was no consistent relationship identified between blood pressure and smoking status (Figs. 5, 6) In addition, smokers with T2DM were likely to be older, had longer duration of smoking history and poorer glycaemic control compared to non-smokers. Smokers have consistently shown lower HDL cholesterol and higher LDL cholesterol compared to non-smokers. Some studies suggested that smokers lose the natural nocturnal dip in blood pressure while some other studies found no diurnal variation in blood pressure between smokers and non-smokers. Further study is needed to understand the exact relationship between smoking and blood pressure in people with diabetes.

#### HbA1c

Meta-analysis of the pooled data showed that the mean difference of HbA1c was −0.61% (95% CI −0.88 to −0.33, p < 0.0001) between non-smokers and smokers (Fig 2). Meta-regression analyses showed that the observed difference in HbA1c levels between smokers and non-smokers was significantly associated with the following factors: (a) mean age of study participants with the difference increasing with mean age (p < 0.001); (b) whether the study was conducted on adults or adolescents participants, with the difference being larger in studies conducted on adults (>22 years) as opposed to adolescents (18–22 years) (p = 0.016); and c) the number of years smoked, with the difference increased as the duration of smoking increased (p = 0.034) (Table 6).

#### Lipid profiles

Meta-analysis of 6 studies with a total sample size of 34,124 demonstrated that the difference in HDL-cholesterol between non-smokers and smokers was 0.12 mmol/l (95% CI 0.08–0.15; p < 0.001). Similarly, the difference in LDL-cholesterol between smokers and non-smokers was 0.11 (95% CI −0.21 to −0.01, p < 0.03) mmol/l. Both these results were statistically significant. Meta-regression examined whether the difference in HDL and LDL cholesterol between smokers and non-smokers was linked with the mean age of study participants, whether the study was conducted on people with T1DM or T2DM, sex of the study participants, study design and duration smoked. No statistically significant correlation was detected with any of the above variables (Tables 7, 8).

#### Blood pressure

Meta-analysis of pooled data from 83,754 participants with type 1 or type 2 diabetes, showed no statistically significant differences in either SBP or DBP between smokers and non-smokers. The mean difference in SBP was −0.34 mm of Hg (95% CI −2.54 to 1.87, p = 0.77) and in DBP was −0.21 mm of Hg (95% CI –1.10 to 0.68, p = 0.64) in non-smokers and smokers, respectively.

Meta-regression analysis, however, showed that the mean difference in SBP was significantly associated with the mean age of the study participants (p = 0.030). Studies with older participants showed a larger difference in SBP between smokers and non-smokers, compared to studies with younger participants. In relation to DBP, the mean difference between smokers and non-smokers was significantly greater in studies that had included adults (>22 years) as opposed to adolescents (18–22 years) (p = 0.041). In addition, difference in DBP was statistically significantly associated with percentage of participants who were male (p = 0.027), suggesting that studies with more male participants showed a larger difference in DBP between smokers and non-smokers (Tables 9, 10).

### Smokers vs. quitters

To compare the outcomes of HbA1c, lipid profiles and blood pressure between smokers and quitters, 5 studies (n = 13,750) (3 cross-sectional, 1 prospective and 1 retrospective design) were analysed. 63.32% of the study participants were continued smokers and 35.06% were quitters. 4 out of 5 studies specified the sex of the study population. In the continued smokers group 57.44% were male and 42.56% were female. In the quitter group, 59.83% were male and 40.17% were female. 97% of the study participants had T2DM and 3% had T1DM.

#### Narrative synthesis

A narrative syntheses of the studies suggest that there was a graded relationship between smoking and quitting on HbA1c. There was a trend of a transient rise in HbA1c following quitting, which lasted from 1 to 3 years depending on the number of cigarettes consumed per day and pack-years smoked. Around 3 years after quitting, continued smokers and quitters had a similar level of HbA1c and around 10 years after quitting, the quitters’ HbA1c was comparable to never-smokers. One of the studies [27] conducted a univariate partial regression efficient and demonstrated that HbA1c declined linearly with the years after smoking cessation (p for trend <0.001).

On the other hand, the improvement in the lipid profile is almost instantaneous after quitting. As early as 3 weeks after quitting, the HDL cholesterol showed a trend to rise in quitters compared to continued smokers. There were insufficient data to make any comments about the outcome of blood pressure following quitting. Meta-analysis was only possible for the outcome of HbA1c between continued smokers and quitters.

#### HbA1c

**Fig. 7:**
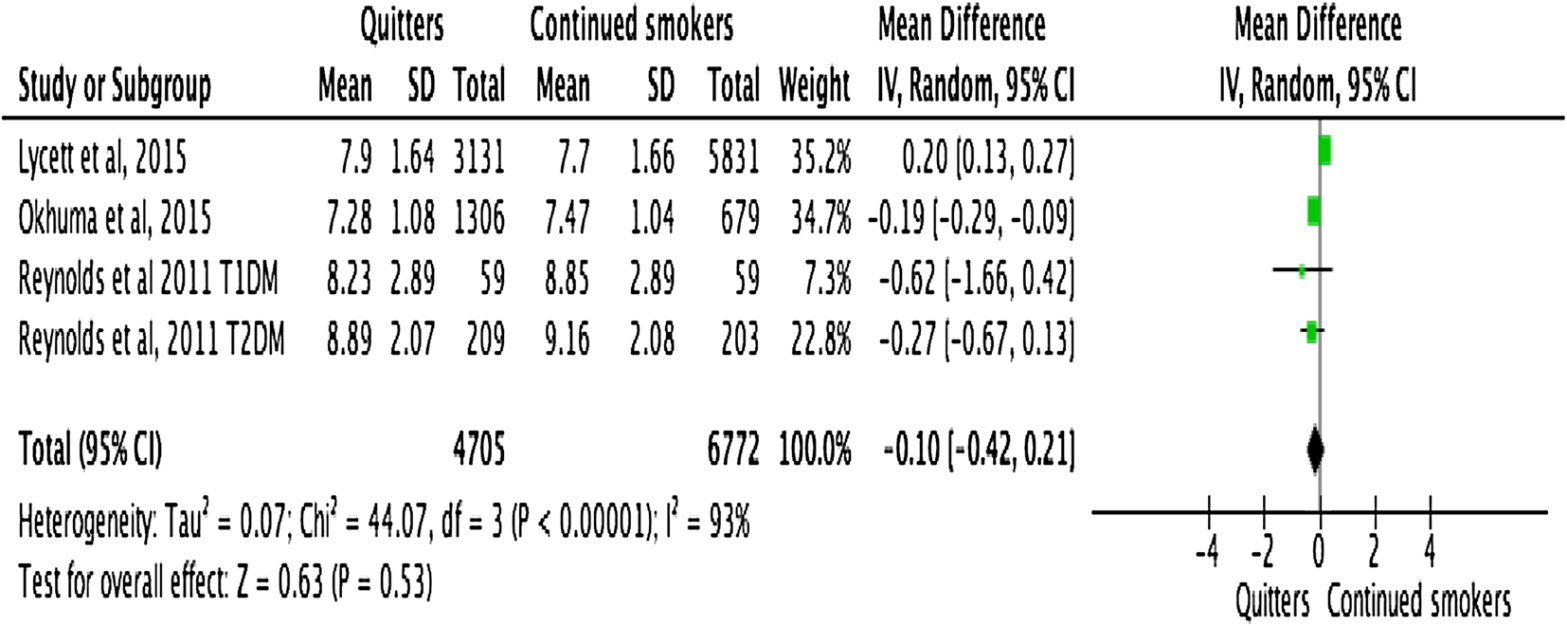
Forest plots. HbA1c (smokers vs. quitters)

Meta-analysis of pooled data from 4 studies showed the difference of HbA1c between quitters and continued smokers was −0.10 (95% CI −0.42 to 0.21, p = *0.53*) (Fig. 7) . This difference was not statistically significant. Meta-regression analysis did not show any statistically significant association between study effect size and mean age of the study population (p = 0.432), whether the study participants were adults (>22 years) or adolescents (18–22 years) (p = 0.39), type of DM (p = 0.64), duration smoked (p = 0.62) and sex of the study participants (p = 0.90). However, there was a statistically significant association between effect size and study design (p = 0.02).

## Discussion

The key findings of the review were non-smokers with diabetes had a lower level of HbA1c −0.61 (95% CI −0.88 to −0.33; p < 0.001), a higher level of HDL cholesterol 0.12 (95% CI 0.08 to 0.15; p < 0.001) and lower level of LDL cholesterol −0.11 (95% CI −0.21 to −0.01; p = 0.03), compared to smokers. However, there was no statistically significant difference in either SBP or DBP between non-smokers and smokers −0.34 (95% CI −2.54 to 1.87; p = 0.77) and −0.21 (95% CI −1.10 to 0.68; p = 0.64), respectively. Univariate adjusted meta-regression analyses revealed that for HbA1c, the difference is significantly associated with the mean age of the study population, whereby studies with an older population resulted in a larger difference in HbA1c between smokers and non-smokers 0.02 (95% CI 0.01, 0.02; p < 0.001). The study effect size was also significantly associated with the duration of smoking 0.021 (95% CI 0.004 to 0.038; p = 0.034) and whether the subjects were adults or adolescents 0.36 (95% CI −0.16 to 0.89; p = 0.016). Univariate adjusted meta-regression analyses revealed that the relationship observed in HDL and LDL cholesterol in non-smokers and smokers was not significantly associated with any of the study level covariates assessed. Whilst meta-regression analysis did not show any significant association between SBP and study level covariates, but difference in DBP was significantly associated with the age of the study participants.

This review did not identify any statistically significant difference in HbA1c between smokers and quitters. The precise effect-size of quitting on lipid profiles and blood pressure could not be accurately delineated, as it was not possible to carry out the meta-analysis due to inadequate number of studies with available data. Conversely, this review did not show the expected reduction in HbA1c after smoking cessation despite overwhelming evidence that the insulin resistance improves after smoking cessation [28, 29].

The major weakness of this review is that it is carried out on observational studies and no temporal relationship can be established. Due to the heterogeneity of study populations, the findings cannot be generalised. The outcome of quitting for less than 12-months is unknown, as this study did not include quitters of less than 12-month duration of abstinence. Despite the outlined weaknesses, this is the first systematic review on this subject, which can be used as a useful tool to raise awareness about the current evidence on this topic of immense public health importance.

The detrimental effects of smoking in diabetes are well documented. It is estimated that cigarettes contain around 4000 chemicals of which, approximately 400 are considered to be harmful [30]. Inhalation is a very efficient method of nicotine delivery, the compound gaining access to the key organs within seconds of administration [31]. After distribution in the circulation, nicotine triggers a cascade of biochemical, hormonal, and metabolic disarray, which appears to be much more pronounced in people with diabetes [32, 33]. A recent study demonstrated that nicotine infusion acutely impairs insulin sensitivity in people with T2DM, but not in healthy subjects suggesting that smoking might affect people with diabetes differently compared to people who do not have diabetes [34]. Several studies have confirmed that smokers with diabetes have a higher HbA1c and atherogenic lipid profiles compared to non-smokers [35, 36]. The relationship of HbA1c and lipid profiles in this study can explain to a large extent the reason why smokers with diabetes have a worse cardiovascular outcome compared to non-smokers.

A number of possible mechanisms, including metabolic deregulation, endothelial dysfunction, and alteration of plasma viscosity by interfering with the coagulation cascade are suggested to explain the association between smoking and diabetes. Nicotine directly inhibits the binding of insulin receptor substrate (IRS) and prevents the activation of intracellular GLUT4, which in turn impedes intracellular glucose transport [37]. This action is followed by a compensatory rise in β-cell insulin secretion as reflected by a higher circulating concentration of c-peptide in smokers, compared to non-smokers [38]. The findings of this review are complementary to the existing knowledge that smokers with diabetes have poorer cardiometabolic profile compared to non-smokers.

However, our current understanding of the impact of quitting on cardiometabolic profile is vague. There are some anecdotal evidence suggesting that a transient rise in HbA1c takes place after smoking cessation [38, 39], while some other studies indicate improved insulin sensitivity as early as 2 weeks after smoking cessation [29]. One of the possible explanations for this paradoxical relationship between the improved insulin sensitivity and a rise in HbA1c, could be a direct blunting effect of smoking on glycosylation of haemoglobin. In a study, researchers measured HbA1c in people without diabetes from the original cohort of Framingham Heart Study and demonstrated that with increasing age and BMI, the HbA1c gradually increased over a period of 4–6 years. On the contrary, smokers did not show any such rise in HbA1c, suggesting that smoking might have a blunting effect on the glycosylation of haemoglobin [40]. It is, therefore, a possibility that after smoking cessation the blunting effect disappears and HbA1c value rises, without any actual deterioration of glycaemic control. Although this study did not show any statistically significant change in HbA1c following quitting for the first 3-years, but it found that if the abstinence continued for 10 years, the HbA1c in quitters was comparable to never smokers. A WHO study showed there was an increase in mortality risk lasting for up to 10 years following quitting, which gradually declined to the level of non-smoker around 10 years after abstinence [30]. This increase in mortality rate may or may not be associated with a transient rise in HbA1c following quitting. As this systematic review showed there was a potential risk of worsening glycaemic control soon after quitting, we recommend that patients should be closely monitored for cardiovascular risk factors following smoking cessation.

It must be emphasized that the harmful effects of smoking are not limited to insulin resistance and poor glycaemic control. It is evident that cigarettes smoking, in addition to metabolic deregulations, plays a crucial role in endothelial dysfunction that contribute to increased cardiovascular mortality in smokers with diabetes. Cigarettes release free radicals into the circulation, which triggers a chronic inflammatory response [31]. Over and above, nicotine and the free radicals alter plasma viscosity by their action on circulating plasminogen activator and natural endothelial vasodilator nitric oxide [32, 33]. The collective outcome is atherosclerosis and a heightened sensitivity of the intrinsic coagulation cascade, which can lead to spontaneous thromboembolism [34]. It is, therefore, not inconceivable that smoking enhances vascular complications in diabetes not only by impaired glucose metabolism but also by several other concomitant vascular perturbations.

Irrespective of the value of HbA1c, the cardiovascular mortality seems to improve after smoking cessation. ADVANCE (action in diabetes and vascular disease: preterax and diamicron modified release controlled evaluation) study has shown, despite moderate weight gain and transient rise in HbA1c, there was a 30% reduction in mortality after smoking cessation [35]. In Nurse’s Health study, the relative risk (RR) of coronary heart disease (CHD) in nurses with type 2 diabetes who had stopped smoking and remained abstinent for 10 years or over was similar to those who never smoked (RR 1.01; 95% CI 0.73–1.38) [6]. After 10 years of abstinence, their HbA1c also dropped at the level of non-smokers.

Dyslipidemia is often associated with diabetes, which seems to be aggravated by smoking. Raised LDL cholesterol is highly atherogenic, particularly with a low level of HDL cholesterol. Nicotine impairs the function of hepatic lipase, which in turns leads to atherogenic dyslipidemia [36]. Several studies have shown marked improvement in lipid profiles following quitting [37, 38]. The HDL cholesterol starts rising as early as within 17 days after quitting [21] and by 48 days there is up to about 15% increase in HDL cholesterol [39]. In this review, we have demonstrated that there was a rise in HDL cholesterol following smoking cessation, consistent with the current evidence.

The influence of smoking and quitting on blood pressure in people with diabetes is not very well studied. Researchers using data from the Health Survey for England for 3 years (1994–1996) examined the relationship between blood pressure and smoking status in people without diabetes [41]. This study was conducted on randomly selected adults (n = 33,860; 47% male) with BP measurements and smoking status (never, current and quitters) and was stratified into younger (age 16–44) and older (>45 years) age groups. After adjusting for age, BMI, social class and alcohol intake older male smokers had higher SBP compared to non-smokers. No such difference was observed (Additional file 3) in SBP in younger group and in DBP in either group. There was no statistically significant difference observed between smokers and quitters for either SBP or DBP. This review did not find any statistically significant difference in blood pressure between smokers, non-smokers and quitters suggesting that the influence of smoking is similar on BP in people with and without diabetes. However, the older smokers may be at a higher risk of vascular complications compared to younger smokers, as their SBP seems to be higher.

## Conclusion

In conclusion, this review demonstrated that smoking negatively impacts upon the cardiometabolic parameters in people with diabetes, which might have a significant detrimental influence on cardiovascular complications. Smoking cessation, on the other hand, improves the cardiometabolic profile particularly by raising cardioprotective HDL cholesterol. However, the benefit of smoking cessation may not be immediately reflected on the HbA1c. The effects of nicotine should be viewed in the bigger context of overall cardiovascular milieu rather than HbA1c alone. Smoking cessation, on the other hand, can cause weight gain and transient rise in HbA1c. Therefore people with diabetes, when they stop smoking, should be followed up very closely and their weight and HbA1c monitored. In addition, at the time of smoking cessation, the healthcare professionals should offer appropriate support to modify lifestyles and consider intensifying pharmacotherapy to address the transient rise of HbA1c.

## Additional files

**Additional file 1:** Database Search - EMBASE/CINAHL

**Additional file 2:** Database search - OVID/Medline

**Additional file 3:** Code for meta-regression

## Abbreviations

DM: diabetes mellitus
T1DM: type 1 diabetes mellitus
T2DM: type 2 diabetes mellitus
HbA1c: glycosylated haemoglobin
LDL-C: low-density lipoprotein cholesterol
HDL-C: high-density lipoprotein cholesterol
SBP: systolic blood pressure
DBP: diastolic blood pressure
