# Supplementary material for: Relationship of cardiometabolic parameters in non-smokers, current smokers, and quitters in diabetes: a systematic review and meta-analysis
Source: Cardiovasc Diabetol. 2016 Nov 24;15:158. doi: 10.1186/s12933-016-0475-5 (PMC5121966; doi:10.1186/s12933-016-0475-5)
Supplement: Supplementary file 1 — Additional file 1: Database Search - EMBASE/CINAHL [file 12933_2016_475_MOESM1_ESM.pdf]

FINAL SEARCH 20/05/16 21

NICE Healthcare Databases Advanced Search

Search history

Select All | Save all | Save selected lines | Delete selected | Remove duplicates | ? | Collapse

Line	Database	Search Term	View Results	
1	CINAHL	exp DIABETES MELLITUS, TYPE 2/ OR exp DIABETES MELLITUS, TYPE 1/	35535	Apply Limits
2	CINAHL	"Diabetes".ti,ab	69041	Apply Limits
3	CINAHL	T2DM.ti,ab	1303	Apply Limits
4	CINAHL	T1DM.ti,ab	288	Apply Limits
5	CINAHL	(IDDM OR insulin AND dependent AND diabetes AND mellitus OR insulin AND treated AND diabetes).ti,ab	2630	Apply Limits
6	CINAHL	(non AND insulin AND dependent AND diabetes AND mellitus).ti,ab	454	Apply Limits
7	CINAHL	(NIDDM OR non AND insulin AND treated AND diabetes).ti,ab	14097	Apply Limits
8	CINAHL	1 OR 2 OR 3 OR 4 OR 5 OR 6 OR 7	77358	Apply Limits
9	CINAHL	(Cigarette AND smoking OR smoking OR tobacco AND addiction AND disorder OR active AND smoking).ti,ab	31984	Apply Limits
10	CINAHL	8 AND 9	3395	Apply Limits
11	CINAHL	("glycaemic control" OR HbA1c OR blood AND glucose).ti,ab	14456	Apply Limits
12	CINAHL	("Lipid profile" OR Low AND density AND lipoprotein OR LDL OR High AND Density AND Lipoprotein OR HDL).ti,ab	10016	Apply Limits
13	CINAHL	("blood pressure" OR BP OR systolic AND blood AND pressure OR SBP OR Diastolic AND Blood AND Pressure OR DBP).ti,ab	28465	Apply Limits
19	EMBASE	exp DIABETES MELLITUS/	701831	Apply Limits
20	EMBASE	("diabetes" OR DM OR T1DM OR Insulin AND dependent AND diabetes).ti,ab	52434	Apply Limits
21	EMBASE	(Type AND 2 AND diabetes AND mellitus OR Type AND 2 AND DM OR T2DM OR non AND insulin AND dependent AND diabetes).ti,ab	15137	Apply Limits
22	EMBASE	(NIDDM OR IDDM).ti,ab	14088	Apply Limits
23	EMBASE	19 OR 20 OR 21 OR 22	710395	Apply Limits
24	EMBASE	("smoking" OR cigarette AND smoking OR tobacco AND exposure).ti,ab	36489	Apply Limits
25	EMBASE	(glycaemic AND control OR HbA1C OR blood AND glucose).ti,ab	152213	Apply Limits
26	EMBASE	23 AND 24 AND 25	113	Apply Limits
27	EMBASE	("lipid profile" OR lipid OR low AND density AND lipoprotein OR LDL OR high AND density AND lipoprotein OR HDL).ti,ab	134650	Apply Limits
28	EMBASE	23 AND 24 AND 27	104	Apply Limits
29	EMBASE	("blood pressure" OR BP OR systolic AND blood AND pressure OR SBP OR diastolic AND blood AND pressure OR DBP).ti,ab	329193	Apply Limits
30	EMBASE	23 AND 24 AND 29	214	Apply Limits
32	EMBASE	23 AND 24 AND 31	449	Apply Limits
33	CINAHL	(smoking AND cessation).ti,ab	7068	Apply Limits
34	CINAHL	(Quit OR give AND up OR abstain).ti,ab	6604	Apply Limits
35	CINAHL	33 OR 34	11540	Apply Limits
36	CINAHL	9 OR 35	35249	Apply Limits
37	CINAHL	8 AND 36	3464	Apply Limits

✓	38	CINAHL	11 AND 37	483	Apply Limits	⊗
✓	39	CINAHL	12 AND 37	500	Apply Limits	⊗
✓	40	CINAHL	13 AND 37	915	Apply Limits	⊗
✓	41	EMBASE	(smoking AND cessation).ti,ab	27657	Apply Limits	⊗
✓	42	EMBASE	(quit OR give AND up OR abstain).ti,ab	27940	Apply Limits	⊗
✓	43	EMBASE	41 OR 42	52924	Apply Limits	⊗
✓	44	EMBASE	24 OR 43	87053	Apply Limits	⊗
✓	45	EMBASE	23 AND 44	3702	Apply Limits	⊗
✓	46	EMBASE	25 AND 45	368	Apply Limits	⊗
✓	47	EMBASE	27 AND 45	295	Apply Limits	⊗
✓	48	EMBASE	29 AND 45	730	Apply Limits	⊗

Combine selected ● AND ○ OR To combine two line numbers using NOT, enter the line numbers in the search box below: for example 1 NOT 2

You are currently searching EMBASE Search another ? » Re-run all line numbers or » Re-run selected line numbers

Search | Limits

Search Keyword:
Enter search words or line numbers in the box below. You may use AND, OR, NOT to combine search words.
Use * to search for word stems, "inverted commas" to search for phrases and round brackets () to nest words.
?

Browse Thesaurus

IN

- ☑ Title and Abstract
- ☐ Any field
- ☐ Publication Type
- ☐ Title
- ☐ Journal Name *
- ☐ Abstract
- ☐ Author *
- ☐ ISSN

More fields

☐ Map to Thesaurus ?

* For Author and Journal name enter search terms in "quotation marks". ?

Search

0 Clipboard is empty 205 Recover searches

Copyright © 2016 National Institute for Health and Care Excellence. All Rights Reserved.

Provided by NI[illegible]
