# Supplementary material for: Relationship of cardiometabolic parameters in non-smokers, current smokers, and quitters in diabetes: a systematic review and meta-analysis
Source: Cardiovasc Diabetol. 2016 Nov 24;15:158. doi: 10.1186/s12933-016-0475-5 (PMC5121966; doi:10.1186/s12933-016-0475-5)
Supplement: Supplementary file 2 — Additional file 2: Database search - OVID/Medline [file 12933_2016_475_MOESM2_ESM.pdf]

FINAL OVID/MEDLINE SEARCH (21.05.16)

Database(s): **Ovid MEDLINE(R) In-Process & Other Non-Indexed Citations and Ovid MEDLINE(R)** 1946 to Present

Search Strategy:

#	Searches	Results	Annotations
1	exp diabetes mellitus/	348228	
2	diabet*.ti,ab,ot.	474512	
3	(IDDM or NIDDM or MODY or T1DM or T2DM or T1D or T2D).ti,ab,ot.	32604	
4	(non insulin* depend* or non insulin* depend* or non insulin ?depend* or non insulin ? depend*).ti,ab,ot.	10681	
5	(insulin* depend* or insulin?depend*).ti,ab,ot.	28385	
6	(Insulin* treated* Type 2 diabetes or insulin treated T2DM or insulin treated T2D).ti,ab,ot.	230	
7	exp smoking cessation/	23079	
8	"Tobacco Use Cessation"/	870	
9	((smoking or tobacco) adj cessation).ti,ab,ot.	19480	
10	((quit* or stop* or cease* or giv*) adj5 smoking).ti,ab.	12513	
11	(smoker* or continued smoker* or persistent smoker*).ti,ab,ot.	68617	
12	"Tobacco Use Disorder"/	9268	
13	(Lipid profile or lipid level or LDL).mp. or low density lipoprotein/ [mp=title, abstract, original title, name of substance word, subject heading word, keyword heading word, protocol supplementary concept word, rare disease supplementary concept word, unique identifier]	94077	
14	HbA1C*.mp. or glycated* haemoglobin*/ [mp=title, abstract, original title, name of substance word, subject heading word, keyword heading word, protocol supplementary concept word, rare disease supplementary concept word, unique identifier]	20239	
15	(High Density Lipoprotein or HDL).mp. [mp=title, abstract, original title, name of substance word, subject heading word, keyword heading word, protocol supplementary concept word, rare disease supplementary concept word, unique identifier]	80124	
16	(Blood pressure or hypertension or systolic blood pressure or diastolic blood pressure*).mp. [mp=title, abstract, original title, name of substance word, subject heading word, keyword heading word, protocol supplementary concept word, rare disease supplementary concept word, unique identifier]	661407	
17	1 or 2 or 3 or 4 or 5 or 6	531587	Any type of DM
18	7 or 8 or 9 or 10 or 11 or 12	90998	Smoking or smoking cessation
19	17 and 18	5533	DM + Smoking (S) or Smoking Cessation (SC)
20	14 and 19	192	DM + S/SC + HbA1C
21	16 and 19	2999	DM + S/SC + BP
22	13 or 15	133312	HDL or LDL or Lipid Profile
23	19 and 22	710	DM + S/SC + Lipid Profile
