# Supplementary material for: Relationship of cardiometabolic parameters in non-smokers, current smokers, and quitters in diabetes: a systematic review and meta-analysis
Source: Cardiovasc Diabetol. 2016 Nov 24;15:158. doi: 10.1186/s12933-016-0475-5 (PMC5121966; doi:10.1186/s12933-016-0475-5)
Supplement: Supplementary file 3 — Additional file 3: Code for meta-regression [file 12933_2016_475_MOESM3_ESM.docx]

--------------------------------------------------------------------------------

name: <unnamed>

log: Z:\Other Files\CLAHRC\Deb Kar\New folder\metaregresults.smcl

log type: smcl

opened on: 16 Aug 2016, 11:16:29

. do "C:\Users\clg13\AppData\Local\Temp\STD00000000.tmp"

. *meta-regression HBA1c never/current

. metareg effHB_NC age, wsse(_se_effHB_NC)

Meta-regression Number of obs = 10

REML estimate of between-study variance tau2 = .008469

% residual variation due to heterogeneity I-squared_res = 69.74%

Proportion of between-study variance explained Adj R-squared = 92.90%

With Knapp-Hartung modification

------------------------------------------------------------------------------

effHB_NC | Coef. Std. Err. t P>|t| [95% Conf. Interval]

-------------+----------------------------------------------------------------

age | .0165339 .0020485 8.07 0.000 .0118101 .0212578

_cons | -1.265896 .1015352 -12.47 0.000 -1.500037 -1.031756

------------------------------------------------------------------------------

. metareg effHB_NC under22, wsse(_se_effHB_NC)

Meta-regression Number of obs = 10

REML estimate of between-study variance tau2 = .09966

% residual variation due to heterogeneity I-squared_res = 95.63%

Proportion of between-study variance explained Adj R-squared = 16.50%

With Knapp-Hartung modification

------------------------------------------------------------------------------

effHB_NC | Coef. Std. Err. t P>|t| [95% Conf. Interval]

-------------+----------------------------------------------------------------

under22 | .3596344 .2288398 1.57 0.155 -.1680711 .8873398

_cons | -1.171983 .382707 -3.06 0.016 -2.054506 -.2894586

------------------------------------------------------------------------------

. metareg effHB_NC diabetestype, wsse(_se_effHB_NC)

Meta-regression Number of obs = 9

REML estimate of between-study variance tau2 = .07319

% residual variation due to heterogeneity I-squared_res = 95.66%

Proportion of between-study variance explained Adj R-squared = 39.75%

With Knapp-Hartung modification

------------------------------------------------------------------------------

effHB_NC | Coef. Std. Err. t P>|t| [95% Conf. Interval]

-------------+----------------------------------------------------------------

diabetestype | .4320316 .2112416 2.05 0.080 -.0674754 .9315386

_cons | -1.260904 .3241538 -3.89 0.006 -2.027406 -.4944017

------------------------------------------------------------------------------

. metareg effHB_NC design, wsse(_se_effHB_NC)

Meta-regression Number of obs = 10

REML estimate of between-study variance tau2 = .1232

% residual variation due to heterogeneity I-squared_res = 98.31%

Proportion of between-study variance explained Adj R-squared = -3.22%

With Knapp-Hartung modification

------------------------------------------------------------------------------

effHB_NC | Coef. Std. Err. t P>|t| [95% Conf. Interval]

-------------+----------------------------------------------------------------

design | -.3279785 .4237343 -0.77 0.461 -1.305112 .6491546

_cons | -.2440426 .479111 -0.51 0.624 -1.348875 .8607894

------------------------------------------------------------------------------

. metareg effHB_NC male, wsse(_se_effHB_NC)

Meta-regression Number of obs = 10

REML estimate of between-study variance tau2 = .09334

% residual variation due to heterogeneity I-squared_res = 98.19%

Proportion of between-study variance explained Adj R-squared = 21.79%

With Knapp-Hartung modification

------------------------------------------------------------------------------

effHB_NC | Coef. Std. Err. t P>|t| [95% Conf. Interval]

-------------+----------------------------------------------------------------

maleper | .0088352 .0047626 1.86 0.101 -.0021473 .0198177

_cons | -1.201532 .3445766 -3.49 0.008 -1.996127 -.4069372

------------------------------------------------------------------------------

. metareg effHB_NC duration, wsse(_se_effHB_NC)

Meta-regression Number of obs = 4

REML estimate of between-study variance tau2 = 0

% residual variation due to heterogeneity I-squared_res = 0.00%

Proportion of between-study variance explained Adj R-squared = 100.00%

With Knapp-Hartung modification

------------------------------------------------------------------------------

effHB_NC | Coef. Std. Err. t P>|t| [95% Conf. Interval]

-------------+----------------------------------------------------------------

duration | .0207644 .0039585 5.25 0.034 .0037322 .0377966

_cons | -1.06308 .1519392 -7.00 0.020 -1.716822 -.4093387

------------------------------------------------------------------------------

.

.

. *meta-regression HDL never/current

. metareg ES_HDL age, wsse(seES_HDL)

numerical derivatives are approximate

nearby values are missing

numerical derivatives are approximate

nearby values are missing

Meta-regression Number of obs = 6

REML estimate of between-study variance tau2 = 0

% residual variation due to heterogeneity I-squared_res = 0.00%

Proportion of between-study variance explained Adj R-squared = 100.00%

With Knapp-Hartung modification

------------------------------------------------------------------------------

ES_HDL | Coef. Std. Err. t P>|t| [95% Conf. Interval]

-------------+----------------------------------------------------------------

age | -.0020339 .0007441 -2.73 0.052 -.0040997 .000032

_cons | .1977952 .0356574 5.55 0.005 .0987943 .2967962

------------------------------------------------------------------------------

. metareg ES_HDL under22, wsse(seES_HDL)

Meta-regression Number of obs = 6

REML estimate of between-study variance tau2 = .001418

% residual variation due to heterogeneity I-squared_res = 52.99%

Proportion of between-study variance explained Adj R-squared = -47.53%

With Knapp-Hartung modification

------------------------------------------------------------------------------

ES_HDL | Coef. Std. Err. t P>|t| [95% Conf. Interval]

-------------+----------------------------------------------------------------

under22 | -.018827 .0449105 -0.42 0.697 -.1435184 .1058644

_cons | .1466923 .064742 2.27 0.086 -.0330603 .326445

------------------------------------------------------------------------------

. metareg ES_HDL diabetestype, wsse(seES_HDL)

Meta-regression Number of obs = 5

REML estimate of between-study variance tau2 = 0

% residual variation due to heterogeneity I-squared_res = 0.00%

Proportion of between-study variance explained Adj R-squared = 100.00%

With Knapp-Hartung modification

------------------------------------------------------------------------------

ES_HDL | Coef. Std. Err. t P>|t| [95% Conf. Interval]

-------------+----------------------------------------------------------------

diabetestype | -.0765843 .0353706 -2.17 0.119 -.1891493 .0359808

_cons | .2387618 .0538389 4.43 0.021 .0674224 .4101011

------------------------------------------------------------------------------

. metareg ES_HDL design, wsse(seES_HDL)

Meta-regression Number of obs = 6

REML estimate of between-study variance tau2 = .001082

% residual variation due to heterogeneity I-squared_res = 44.79%

Proportion of between-study variance explained Adj R-squared = -12.57%

With Knapp-Hartung modification

------------------------------------------------------------------------------

ES_HDL | Coef. Std. Err. t P>|t| [95% Conf. Interval]

-------------+----------------------------------------------------------------

design | .0352807 .0569325 0.62 0.569 -.1227891 .1933505

_cons | .0794386 .0680363 1.17 0.308 -.1094603 .2683376

------------------------------------------------------------------------------

. metareg ES_HDL male, wsse(seES_HDL)

numerical derivatives are approximate

nearby values are missing

numerical derivatives are approximate

nearby values are missing

Meta-regression Number of obs = 6

REML estimate of between-study variance tau2 = 0

% residual variation due to heterogeneity I-squared_res = 0.00%

Proportion of between-study variance explained Adj R-squared = 100.00%

With Knapp-Hartung modification

------------------------------------------------------------------------------

ES_HDL | Coef. Std. Err. t P>|t| [95% Conf. Interval]

-------------+----------------------------------------------------------------

maleper | -.0014512 .0005321 -2.73 0.053 -.0029284 .0000261

_cons | .2335497 .0483728 4.83 0.008 .0992452 .3678543

------------------------------------------------------------------------------

. metareg ES_HDL duration, wsse(seES_HDL)

insufficient observations

r(2001);

end of do-file

r(2001);

. log close

name: <unnamed>

log: Z:\Other Files\CLAHRC\Deb Kar\New folder\metaregresults.smcl

log type: smcl

closed on: 16 Aug 2016, 11:17:23

--------------------------------------------------------------------------------
